# Associations between antiretroviral therapy-related experiences and mental health status among people living with HIV in China: a prospective observational cohort study

**DOI:** 10.1186/s12981-021-00370-y

**Published:** 2021-09-09

**Authors:** Jinzhao Xie, Zixin Wang, Quanmin Li, Qiangsheng He, Guohong Xu, Yonghong Li, Kai Zhou, Linghua Li, Jing Gu

**Affiliations:** 1 Department of Medical Statistics, School of Public Health, Sun Yat-Sen University, Guangzhou, 510080 Guangdong China; 2 JC School of Public Health and Primary Care, The Chinese University of Hong Kong, Hong Kong, China; 3 CUHK Shenzhen Research Institute (SZRI), Shenzhen, 518172 Guangdong China; 4 Department of Infectious Diseases, Guangzhou Eighth People’s Hospital, Guangzhou Medical University, Guangzhou, 510080 Guangdong China; 5 Clinical Research Center, The Seventh Affiliated Hospital, Sun Yat-Sen University, Shenzhen, 518107 Guangdong China; 6 Dean’s Office, Hospital of Stomatology, Sun Yat-Sen University, Guangzhou, 510055 Guangdong China; 7 Sun Yat-Sen Global Health Institute, School of Public Health and Institute of State Governance, Guangzhou, 510080 Guangdong China

**Keywords:** HIV, Antiretroviral therapy, Mental health, China

## Abstract

**Background:**

Mental health problems (e.g., depression and anxiety) are among the most commonly reported comorbidities of HIV. Antiretroviral therapy (ART) coverage has increased sharply. The purposes of this prospective cohort study were to investigate the ART-related experiences and whether they were associated with mental health problems among a sample of people living with HIV undergoing ART in China.

**Methods:**

The participants were 400 people living with HIV who had started ART for the first time in Guangzhou city. They were followed-up 1-year after ART initiation. Probable depression and moderate/severe anxiety were measured at baseline and Month 12, while experiences related to ART (e.g., side effects and regained self-confidence) were measured at Month 6. Univariate and multivariate logistic regressions were used to explore the associations between baseline characteristics, ART-related experiences and mental health status.

**Results:**

Among the 300 participants (75.0%) who completed all three surveys, a significant decline in prevalence of probable depression (23.0% at baseline vs. 14.0% at Month 12, *P* = 0.002) and moderate/severe anxiety (14.7% at baseline vs. 8.7% at Month 12, *P* = 0.023) was observed during the follow-up period. After adjustment for mental health status and potential confounders at baseline, a number of ART-related experiences at Month 6 were associated with probable depression and/or moderate/severe anxiety measured at Month 12. Improved physical health, relationships with sexual partners, and self-confidence were associated with decreased mental health issues, while the side effects of ART, AIDS-related symptoms, and inconvenience in daily life due to ART use were associated with increased mental health issues.

**Conclusions:**

ART-related experiences were associated with mental health problems, tailored mental health promotion interventions targeting these experiences are needed.

## Background

Antiretroviral therapy (ART) has increased the life expectancy of people living with HIV (PLWH), allowing them to live longer with this chronic medical condition [1, 2]. In line with the World Health Organization (WHO), China renewed its national ART guidelines in 2016 [3], and ART coverage increased from 67% in 2015 to 80% in 2017 [4]. After entering the “treat all” era [5], ART coverage and effectiveness has increased sharply across countries [6, 7]. Recently, researchers have shifted their focus toward the next targets following viral suppression. The UNAIDS added a fourth 90 target (i.e., ensuring that 90% of people with viral load suppression have good health-related quality of life) to the existing agenda [8].

Mental health problems (e.g., depression and anxiety) are among the most commonly reported comorbidities of HIV [9–11], affecting quality of life and wellbeing among PLWH [12, 13]. Evidence suggests that depression and anxiety are associated with low medical adherence [14], worse retention in HIV care [15, 16], and poor HIV-related outcomes (e.g., quality of life) [17, 18]. A meta-analysis has shown an overall prevalence of depression and anxiety of 33.6% and 28.4%, respectively, among PLWH on ART [19]. In China, the prevalence of depression was reported as about 40% among PLWH on ART [20, 21]. Given these findings, the prevention and treatment of mental health problems may be an important element of HIV treatment and care.

A growing body of literature has demonstrated improved mental health status among PLWH after ART initiation. A longitudinal study in the United States showed that, compared to pre-treatment levels, the prevalence of depression decreased from 30 to 21% after 1 year on ART [22]. It is possible that the benefits of ART are mediated via a reduction in inflammatory pathways that affect the risk of depression, such as the kynurenine pathway or tryptophan catabolism [23]. On the other hand, ART may also indirectly improve mental health status among PLWH by increasing access to health care services, psychological services and community support [24]. Although previous studies have identified a number of factors that are associated with mental health among PLWH on ART (e.g. gender [25], personal income level [26], living arrangements [27], and family support [21]), most of these studies were cross-sectional and ignored therapy-related experiences.

ART requires lifelong treatment, which brings both positive and negative experiences for users. On the one hand, ART brings significant clinical benefits for PLWH, such as improving physical health status, facilitating immune system recovery (e.g., higher CD4 cell counts), and slowing disease progression [28, 29]. There is also evidence that viral suppression by ART greatly reduces the risk of transmitting HIV to sexual partners [1, 2], potentially improving relationships between PLWH and their sexual partners. On the other hand, although advances in ART have largely reduced its side effects, recent studies have shown that about 25.0–53.3% of PLWH still experience severe side effects in their first year after ART initiation [30, 31]. Moreover, the daily regimen of ART is inconvenient for PLWH. For example, 25% of PLWH in eight high-income countries agreed that being tied to daily medication limited their day-to-day life, and 29% felt stressed by the need to take their medication at the right time every day [32]. These experiences related to ART may also influence mental health among PLWH. Previous studies found that the side effects of ART, lowered CD4 cell counts, and AIDS-related symptoms after ART initiation were associated with depression and anxiety [20, 26, 27]. However, these studies were limited by their cross-sectional nature and the lack of inclusion of potentially relevant experiences of ART.

As far as we know, no longitudinal study has investigated ART-related experiences and whether they were associated with mental health problems among PLWH. To address these gaps, this prospective cohort study measured ART-related experiences at Month 6 and mental health problems at Month 12 among a sample of PLWH in China who had initiated ART for the first time, and investigated whether ART-related experiences (Month 6) were associated with mental health problems (Month 12) after considering potential confounders.

## Methods

### Study population and procedure

This prospective cohort study was conducted in Guangzhou, China, from June 2016 to May 2018. Face-to-face interviews were conducted at baseline (the first day of ART admission), and at Month 6 and Month 12 after ART admission. The inclusion criteria were that the subjects were (1) aged 18 years or above, (2) HIV seropositive, (3) initiating ART for the first time, and (4) willing to attend surveys at baseline, Month 6, and Month 12. Those who had major psychiatric illnesses (schizophrenia or bipolar disorder) or could not communicate with the interviewers were excluded.

The participants were recruited from the ART clinic of Guangzhou Eighth People’s Hospital in Guangzhou. The Guangzhou Eighth People’s Hospital was the first ART centre in Guangdong province and has provided ART to over 10,000 PLWH since 2004 [33]. During the recruitment period (June 2016 to May 2017), trained nurses screened the new ART users for eligibility and referred those who met the criteria to the research team. The trained interviewers then reconfirmed the participants’ eligibility, explained the details of the study, and assured them that refusal would not affect their right to use any related services and that they could quit the study at any time without being questioned. After providing written informed consent, the participants completed a self-administered questionnaire which took about 15 min. They were invited to visit the hospital again to complete two other self-administered questionnaires after 6 and 12 months. On completion of each of the three surveys, the participants received a souvenir (worth 15 RMB or 2 USD) in appreciation of their time. Ethical approval for the study was obtained from the Institutional Review Board (IRB) of the School of Public Health, Sun Yat-sen University, Guangzhou, China (No: 2016-003).

Of the 480 prospective participants approached, 425 passed the eligibility screening. Of these, 400 provided written informed consent and completed the baseline survey (response rate: 94.1%, 400/425), among whom 353 (88.3%, 353/400) completed the Month 6 follow-up survey, and 300 (75.0%, 300/400) also completed the Month 12 survey. Of the participants who did not complete both follow-up surveys (n = 100), 12 were lost to clinic follow-up (didn’t come back since the last visit), 11 were transferred to other ART clinics, 2 were died, 4 refused to participate in the follow-up surveys, and 71 didn’t show up during the appointed follow-up period but remained in the treatment.

### Measurements

#### Mental health problems measured at baseline and Month 12

Depressive symptoms were measured by the validated Chinese version of the Patient Health Questionnaire (PHQ-9) [34, 35]. The PHQ-9 has been widely used to screen for depression in different Chinese populations [34, 35], including PLWH [36], and has acceptable reliability and validity. The scale contains nine items regarding the frequency of certain negative feelings over the past 2 weeks (response categories: 0 = never, 1 = sometimes, 2 = often, 3 = almost every day). The total scores ranged from 0 to 27, and a higher score indicated more severe depression. A score of ≥ 10 was used to define probable depression [22, 26].

Generalized anxiety disorder was measured by the validated Chinese version of the Generalized Anxiety Disorder Scale (GAD-7), which has shown acceptable reliability and validity in prior research [37]. The scale contains 7 items, with a score range of 0–21, a higher score representing greater anxiety. In this study, a score of ≥ 10 was used to define moderate/severe anxiety [38].

#### Experiences related to ART measured at Month 6

A panel of HIV epidemiologists, behavioural health researchers, nurses, and physicians from the ART clinic was formed to develop the questions regarding patients’ experiences related to ART. The questions were then pilot tested among 15 PLWH who were currently on ART. Based on their feedback, the experience questions were revised to form the experiences measurement, which included four positive and four negative experiences.

Four items measured positive experiences related to ART. The participants were asked to report whether they had had the following experiences during the past 6 months: (1) a rise in CD4 cell count, (2) improvement in physical health, (3) improvement in relationships with sexual partners, and (4) regained self-confidence (response categories: 1 = yes, 0 = no). Negative experiences related to ART in the past 6 months were also measured by four items: (1) side effects of ART, (2) AIDS-related symptoms, (3) inconvenience of ART use in daily life, and (4) exposure of HIV-positive status due to ART use (response categories: 1 = yes, 0 = no).

#### Baseline characteristics

The participants were asked about their *background characteristics*, including age, sex, education, current marital status, monthly personal income, employment status, and city of permanent residence. *HIV/AIDS-related characteristics* were also recorded, including AIDS-related symptoms, CD4 cell count at the most recent test, route of HIV transmission (sexual behaviour with same-sex partner, sexual behaviour with opposite-sex partner, intravenous drug use, mother-to-child transmission, blood transfusion, occupational exposure, or not sure), and ART regime.

*Psychosocial status* including social support and social stigma was also measured at baseline. Social support was measured by the validated Chinese version of the Multidimensional Scale of Perceived Social Support (MSPSS) [39, 40]. The MSPSS contains 12 items and 3 dimensions including family, friends, and significant others. The score range for each dimension is 1–7, with a higher score indicating better support. We used the social stigma module of the Chinese Courtesy Stigma Scales (CSSSs) to measure the social stigma patients experienced [41]. The scale contains 13 items, with a score range of 13–52, a higher score representing greater stigma.

### Statistical analysis

The baseline characteristics of those who completed all follow-up surveys and those who did not were compared using a chi-square test (for categorical variables), independent samples t-test (for continuous variables with normal distribution), or non-parametric test (for ranked variables and continuous with non-normal/skewed distribution). Further analysis was performed among those who completed all three surveys: baseline, Month 6, and Month 12 (n = 300).

The depression and anxiety status were presented using both scores of assessments (continuous variables) and clinically defined thresholds (categorical variables). The difference in mental health problems between baseline and Month 12 was tested by the Wilcoxon signed rank test (for scores) and the paired-sample Chi-square test (for categorical mental health statuses). Associations between baseline characteristics and probable depression and moderate/severe anxiety measured at Month 12 were described by the odds ratio (OR) obtained from univariate logistic regression models. Multivariate stepwise logistic regression models were used to identify baseline factors that independently affected mental health status at Month 12. To test whether ART-related experiences measured at Month 6 were associated with future mental health problems (probable depression and moderate/severe anxiety measured at Month 12), both univariate and adjusted logistic regression models were applied, adjusted for baseline variables with *P* < 0.1, plus baseline mental health status. R software version 4.0.2 was used for the data analysis, and *P* < 0.05 was considered statistically significant.

## Results

### Baseline characteristics of participants

Of the 400 participants, most were male (92.2%), more than 25 years old (79.6%), currently single (55.8%), and non-permanent residents of Guangzhou (77.7%). About 60% of the participants reported that they became infected through sexual behaviour with a same-sex partner. Over 80% used a combination of tenofovir, lamivudine, and efavirenz (84.0%) as their ART regimen. At baseline, the median and lower and upper quartiles [M (Q_1_, Q_2_)] of depressive and anxious scores were 7.00 (4.00, 10.00) and 6.00 (2.00, 7.00) respectively, the prevalence of probable depression and moderate/severe anxiety was 26.0% and 15.0%, respectively.

Those who did not complete both follow-up surveys were more likely to be older, have a lower or no fixed monthly personal income, have probable depression at baseline, and not have been infected through sexual behaviour with a same-sex partner (Table 1).

**Table 1:** Baseline characteristics of all participants and by follow up status

Variables	All participants (*n* = 400)	Participants who completed follow-up surveys (*n* = 300)	Participants who did not complete follow-up surveys (*n* = 100)	*P*
	*n* (%)	*n* (%)	*n* (%)	
Background characterist﻿ics				
Age (years) ($\overline{x} \pm {\text{s}}$)	32.62 ± 9.72	31.88 ± 9.03	34.78 ± 11.33	0.010
18–	82 (20.4)	65 (21.7)	17 (17.0)	0.020
25–	171 (42.8)	133 (44.3)	38 (38.0)	
35–	92 (23.0)	70 (23.3)	22 (22.0)	
45–	55 (13.8)	32 (10.7)	23 (23.0)	
Sex				0.105
Female	31 (7.8)	19 (6.3)	12 (12.0)	
Male	369 (92.2)	281 (93.7)	88 (88.0)	
Education				0.082
Primary school and below	133 (33.2)	91 (30.3)	42 (42.0)	
Junior or senior high	102 (25.6)	82 (27.4)	20 (20.0)	
College and above	165 (41.2)	127 (42.3)	38 (38.0)	
Current marital status				0.757
Married or cohabiting with a partner	136 (34.0)	104 (34.7)	32 (32.0)	
Single	223 (55.8)	167 (55.6)	56 (56.0)	
Other	41 (10.2)	29 (9.7)	12 (12.0)	
Monthly personal income in RMB (USD)				0.005
No fixed income	79 (19.8)	50 (16.7)	29 (29.0)	
< 3000 (< 433 USD)	97 (24.2)	70 (23.3)	27 (27.0)	
3000-(433-USD)	127 (31.8)	108 (36.0)	19 (19.0)	
5000-(722-USD)	97 (24.2)	72 (24.0)	25 (25.0)	
Employment status				0.814
Full-time	238 (59.5)	177 (59.0)	61 (61.0)	
Part-time/unemployed/retired/student	162 (40.5)	123 (41.0)	39 (39.0)	
City of permanent residence				0.627
Guangzhou	89 (22.3)	69 (23.0)	20 (20.0)	
Other cities	311 (77.7)	231 (77.0)	80 (80.0)	
HIV/AIDS-related characteristics				
Presence of AIDS-related symptoms				0.343
No	246 (61.5)	189 (63.0)	57 (57.0)	
Yes	154 (38.5)	111 (37.0)	43 (43.0)	
CD4 cell count (cell/mm^3^)				0.304
< 200	112 (28.0)	78 (26.0)	34 (34.0)	
200–	173 (43.2)	135 (45.0)	38 (38.0)	
350–	75 (18.8)	59 (19.7)	16 (16.0)	
500–	40 (10.0)	28 (9.3)	12 (12.0)	
Route of HIV transmission				0.013
Sexual behavior with same-sex partner	236 (59.0)	191 (63.7)	45 (45.0)	
Sexual behavior with opposite-sex partner	89 (22.2)	59 (19.7)	30 (30.0)	
Other routes^a^	21 (5.2)	14 (4.6)	7 (7.0)	
Not sure	54 (13.6)	36 (12.0)	18 (18.0)	
ART regimen				0.431
Tenofovir, lamivudine, and efavirenz	336 (84.0)	255 (85.0)	81 (81.0)	
Other	64 (16.0)	45 (15.0)	19 (19.0)	
Psychosocial status ($\overline{x} \pm {\text{s}}$)				
Social support (range 1–7)				
Family	4.80 ± 1.45	4.79 ± 1.45	4.83 ± 1.45	0.807
Friends	4.86 ± 1.37	4.90 ± 1.35	4.74 ± 1.42	0.316
Significant others	5.29 ± 1.22	5.34 ± 1.19	5.13 ± 1.28	0.129
Social stigma (range 13–52)	29.80 ± 6.77	29.86 ± 6.68	29.65 ± 7.04	0.792
Mental health problems [M (Q_1_, Q_2_)]				
Probable depression (range 0–27)	7.00 (4.00, 10.00)	7.00 (4.00, 9.00)	7.00 (3.00, 10.00)	0.504
No (PHQ-9 score < 10)	296 (74.0)	231 (77.0)	65 (65.0)	0.025
Yes (PHQ-9 score ≥ 10)	104 (26.0)	69 (23.0)	35 (35.0)	
Moderate/severe anxiety (range 0–27)	6.00 (2.00, 7.00)	6.00 (2.00, 7.00)	4.00 (2.00, 8.00)	0.833
No (GAD-7 score < 10)	340 (85.0)	256 (85.3)	84 (84.0)	0.872
Yes (GAD-7 score ≥ 10)	60 (15.0)	44 (14.7)	16 (16.0)	

M (Q1, Q2): Median (lower quartile, upper quartile)

^a^Intravenous drug use, mother-to-child transmission, blood transfusion, occupational exposure

### Changes in mental health problems from baseline to Month 12

Among participants who completed all three surveys (n = 300), the prevalence of probable depression declined from 23.0% at baseline to 14.0% at Month 12 (paired *χ*^*2*^ = 9.80, *P* = 0.002). The prevalence of moderate/severe anxiety was 14.7% at baseline and 8.7% at Month 12 (paired *χ*^*2*^ = 5.16, *P* = 0.023). The depressive scores declined from 7.00 (4.00, 9.00) [M (Q_1_, Q_2_)] at baseline to 5.00 (1.00, 8.00) [M (Q_1_, Q_2_)] at Month 12 (*P* < 0.001). The anxious scores were 6.00 (2.00, 7.00) [M (Q_1_, Q_2_)] at baseline and 3.00 (0.00, 7.00) [M (Q_1_, Q_2_)] at Month 12 (*P* < 0.001).

### Experiences related to ART measured at Month 6

Among participants who completed all three surveys (n = 300), most reported a rise in CD4 cell count (90.0%), improvement in physical health status (80.3%), and regained self-confidence (74.7%). Some also reported improvement in relationships with sexual partners since ART initiation (21.7%). On the negative side, 76.3% experienced inconvenience in daily life due to ART use and 50.7% suffered side effects of ART. Some participants also reported AIDS-related symptoms (18.3%) and exposure of their HIV-positive status due to ART use (22.7%).

### Associated factors of probable depression and moderate/severe anxiety measured at Month 12

Participants who were female, received less support from friends and significant others, perceived greater social stigma, and had probable depression and moderate/severe anxiety at baseline were more likely than others to have probable depression at Month 12. Participants who were younger, had lower monthly personal income, received less support from friends, and perceived greater social stigma at baseline were more likely to have moderate/severe anxiety at Month 12 (Table 2). Stepwise regression models showed that sex, support from family and significant others, and probable depression at baseline affected depression at Month 12 independently. Support from friends and social stigma were independent factors associated with moderate/severe anxiety at Month 12.

**Table 2:** Associations between baseline variables and mental health problems at Month 12 (among those who completed all three surveys: baseline, Month 6, and Month 12, n = 300)

Variables	Probable depression		Moderate/severe anxiety	
	Row%	OR_u_ (95% CI)	Row%	OR_u_ (95% CI)
Background characteristics				
Age (years)				
18–	16.9	1.00	13.8	1.00
25–	9.8	0.53 (0.22, 1.28)	5.3	0.35 (0.12, 0.97)*
35–	17.1	1.02 (0.41, 2.53)	11.4	0.80 (0.28, 2.24)
45–	18.8	1.13 (0.36, 3.33)	6.2	0.41 (0.06, 1.74)
Sex				
Female	36.8	1.00	21.1	1.00
Male	12.5	0.24 (0.09, 0.69)**	7.8	0.32 (0.10, 1.19)^†^
Education				
Primary school and below	16.5	1.00	8.8	1.00
Junior or senior high	14.6	0.87 (0.37, 1.98)	13.4	1.61 (0.62, 4.36)
College and above	11.8	0.68 (0.31, 1.48)	5.5	0.61 (0.20, 1.75)
Current marital status				
Married or cohabiting with a partner	14.4	1.00	10.6	1.00
Single	14.4	1.00 (0.50, 2.04)	7.8	0.71 (0.31, 1.69)
Other	10.3	0.68 (0.15, 2.28)	6.9	0.63 (0.09, 2.52)
Monthly personal income in RMB (USD)				
No fixed income	18.0	1.00	14.0	1.00
< 3000 (< 433 USD)	17.1	0.94 (0.37, 2.50)	12.9	0.91 (0.31, 2.71)
3000-(433-USD)	14.8	0.79 (0.33, 2.01)	7.4	0.49 (0.17, 1.48)
5000-(722-USD)	6.9	0.34 (0.10, 1.05)^†^	2.8	0.18 (0.03, 0.77)*
Employment status				
Full-time	13.0	1.00	7.9	1.00
Part-time/unemployed/retired/student	15.4	1.22 (0.63, 2.36)	9.8	1.26 (0.55, 2.83)
City of permanent residence				
Guangzhou	8.7	1.00	5.8	1.00
Other cities	15.6	1.94 (0.83, 5.3)	9.5	1.71 (0.63, 6.01)
HIV/AIDS-related characteristics				
Presence of AIDS-related symptoms				
No	14.8	1.00	9.5	1.00
Yes	12.6	0.83 (0.41, 1.64)	7.2	0.74 (0.29, 1.71)
CD4 cell count (cell/mm^3^)				
< 200	12.8	1.00	10.3	1.00
200–	15.6	1.25 (0.57, 2.92)	8.9	0.85 (0.34, 2.27)
350–	10.2	0.77 (0.25, 2.21)	6.8	0.64 (0.16, 2.13)
500–	17.9	1.48 (0.42, 4.63)	7.1	0.67 (0.10, 2.90)
Route of HIV transmission				
Sexual behavior with same-sex partner	12.0	1.00	8.4	1.00
Sexual behavior with opposite-sex partner	15.3	1.31 (0.55, 2.94)	6.8	0.80 (0.22, 2.28)
Other routes^#^	14.3	1.22 (0.18, 4.84)	7.1	0.84 (0.04, 4.66)
Not sure	22.2	2.09 (0.81, 4.98)	13.9	1.76 (0.55, 4.88)
ART regimen				
Tenofovir, lamivudine, and efavirenz	13.7	1.00	8.2	1.00
Other	15.6	1.16 (0.45, 2.66)	11.1	1.39 (0.45, 3.65)
Psychosocial status				
Social support ($\overline{x} \pm {\text{s}}$) (range 1–7)				
Family	N.A	0.98 (0.79, 1.24)	N.A	0.83 (0.64, 1.09)
Friends	N.A	0.71 (0.57, 0.90)**	N.A	0.61 (0.46, 0.81)***
Significant others	N.A	0.71 (0.55, 0.91)**	N.A	0.76 (0.56, 1.03)^†^
Social stigma ($\overline{x} \pm {\text{s}}$) (range 13–52)	N.A	1.06 (1.01, 1.12)*	N.A	1.08 (1.02, 1.15)**
Mental health problems				
Probable depression (PHQ-9 score ≥ 10)	30.4	4.37 (2.21, 8.69)***	14.5	2.28 (0.95, 5.22)^†^
Moderate/severe anxiety (GAD-7 score ≥ 10)	25.0	2.42 (1.07, 5.17)*	15.9	2.36 (0.87, 5.79)^†^

*N.A.* Not applicable

^#^Intravenous drug use, mother-to-child transmission, blood transfusion, occupational exposure

^†^ 0.05 < *P* < 0.1; * *P* < 0.05; ** P < 0.01; *** *P* < 0.001

After adjustment for baseline variables with *P* < 0.1 in the univariate analysis plus baseline depression and anxiety status, those who reported improved physical health at Month 6 were less likely to have moderate/severe anxiety (OR_a_ = 0.31, 95% CI 0.12 to 0.84) at Month 12. Those reporting improved relationships with sexual partners at Month 6 were less likely to have either probable depression (OR_a_ = 0.25, 95% CI 0.06 to 0.73) or moderate/severe anxiety at Month 12 (OR_a_ = 0.17, 95% CI 0.02 to 0.71), and those reporting regained self-confidence were also less likely to have probable depression (OR_a_ = 0.40, 95% CI 0.19 to 0.87). AIDS-related symptoms were associated with probable depression (OR_a_ = 3.15, 95% CI 1.30 to 7.57) at Month 12. Side effects of ART and inconvenience in daily life due to ART use were associated with moderate/severe anxiety at Month 12 (Table 3).

**Table 3:** Associations between ART experiences at Month 6 and mental health problems at Month 12 (among those who completed all three surveys: baseline, Month 6, and Month 12, n = 300)

**Experiences during ART**	Probable depression		Moderate/severe anxiety	
	OR_u_ (95% CI)	OR_a_^a^ (95% CI)	OR_u_ (95% CI)	OR_a_^b^ (95% CI)
Positive experiences				
Increased CD4 cell count	1.52 (0.50, 6.57)	–	0.84 (0.27, 3.69)	–
Improved physical health	0.42 (0.21, 0.88)*	0.63 (0.28, 1.47)	0.29 (0.13, 0.68)**	0.31 (0.12, 0.84)*
Improved relationships with sexual partners	0.34 (0.10, 0.89)*	0.25 (0.06–0.73)*	0.28 (0.04, 0.98)^†^	0.17 (0.02, 0.71)*
Regained self-confidence	0.30 (0.16, 0.60)***	0.40 (0.19–0.87)*	0.36 (0.16, 0.82)*	0.47 (0.19, 1.21)
Negative experiences				
Side effects of ART	2.16 (1.10, 4.40)*	2.07 (0.99–4.51)^†^	3.59 (1.48, 10.05)**	3.49 (1.35, 10.38)*
AIDS-related symptoms	2.00 (0.92, 4.14)^†^	3.15 (1.30–7.57)**	2.15 (0.84, 5.09)^†^	2.82 (0.96, 8.04)^†^
Inconvenience in daily life due to ART use	1.37 (0.63, 3.33)	–	4.04 (1.16, 25.54)^†^	5.74 (1.36, 41.92)*
Exposure of HIV-positive status due to ART use	0.65 (0.25, 1.45)	–	1.29 (0.48, 3.08)	–

– Not significant in univariate analysis

^a^OR_a_: Adjusted odds ratios, adjustment for mental health status measured at baseline (probable depression and moderate/severe anxiety) and baseline background variables with *P* < 0.1 (sex, monthly personal income, friend support, significant other support, and social stigma)

^b^OR_a_: Adjusted odds ratios, adjustment for mental health status measured at baseline (probable depression and moderate/severe anxiety) and baseline background variables with *P* < 0.1 (age group, sex, monthly personal income, friend support, significant other support, and social stigma)

^†^ 0.05 < *P* < 0.1; * *P* < 0.05; ** P < 0.01; *** *P* < 0.001

## Discussion

Our research is one of the few longitudinal studies to investigate the association between ART-related experiences and mental health problems among PLWH who have recently started ART. In our study, the prevalence of probable depression and moderate/severe anxiety both at baseline and at Month 12 was lower than that reported by meta-analyses targeting PLWH in China [9, 42]. This discrepancy may be attributable to the broad range of measurements used in different studies and to differences in the populations studied (e.g., gender identity, age, region, treatment status) [20, 21, 43]. In studies using the same measurements as in our study, the prevalence of depression was 39.3% among newly diagnosed PLWH [44], and the prevalence of anxiety was 64.3% among those who had newly initiated ART [45]. Similar to previous longitudinal studies [22, 46], our study showed a significant decline in the prevalence of mental health problems at Month 12 compared to pre-treatment. Among those who remained in the cohort, baseline mental health status was a significant factor of mental health problems 1 year after starting ART. Screening for mental health problems and related care is greatly warranted for PLWH who were new to ART.

This study provides healthcare workers with insights for promoting mental health among PLWH who are starting ART, especially regarding segmentation. According to social marketing approaches, careful segmentation improves the effectiveness of health promotion programs [47]. Our study showed the characteristics of those who were more likely to drop out from the study cohort. As a significant of these participants also dropped out from ART, it indicated the need of more proactive intervention to reduce loss to follow-up among those who were older, had a lower or unfixed monthly income, had probable depression, and were infected through heterosexual behaviours and intravenous drug use at the beginning of ART program. Similar to previous studies, females are found to be at higher risk of mental health problems after receiving ART [48, 49]. As female PLWH may suffer higher levels of internalized stigma and experience more social stigma than male patients [48, 49], more attention should be focused on female PLWH in future mental health promotion programs. Furthermore, PLWH without fixed incomes or with relatively low incomes should be prioritized in future programs, as they not only tend to drop out of programs but also to have a higher prevalence of mental health problems 1 year after receiving ART. Although ART is provided for free to all PLWH in China [50], PLWH still need to pay for compulsory physical examinations every 3 months. This financial burden may become a stressor, especially for those with lower incomes.

Similar to the findings of cross-sectional studies [10, 51], we found that stronger social support and lower perceived social stigma at baseline were protective factors for mental health problems. A study among Chinese PLWH also showed that family and social networks, and the trust/intensity of relationships with family members and others played important roles in mental wellbeing [52]. In addition, studies have shown that living in a trusting social environment with lower stigma toward PLWH alleviates daily stressors and protects against mental health problems [52–54]. On the one hand, strategies to encourage and teach PLWH to communicate with their family/friends for support are needed [55]. On the other hand, public health education programs aimed at reducing discrimination against PLWH may help provide PLWH on ART with more social support to promote their mental health.

Our findings highlight the influence of ART-related experiences on mental health problems among PLWH, as a number of positive/negative experiences were significantly or marginally significantly associated with probable depression and/or moderate/severe anxiety after controlling for potential baseline confounders. Consistent with previous findings, side effects of ART may trigger depressive and anxiety symptoms [20, 21, 27]. About half of our participants experienced some side effects at Month 6. These side effects bring physical discomfort to PLWH on ART, interrupting their normal daily and social functioning and reducing quality of life [31]. Thus, the development of effective interventions to facilitate self-management of side effects is greatly needed. Participants with AIDS-related symptoms were more likely to develop probable depression, in line with previous studies [26, 27]. A review has reported a significant association between depressive symptoms and HIV progression [56]. The significant association may be due to the chronic impact of HIV on immune and disease-related parameters, which in turn may lead to depressive symptoms among PLWH on ART [57]. We found that about three quarters of the participants reported inconvenience in daily life due to ART use, and this was associated factor of moderate/severe anxiety. Taking ART every day may be a reminder of their HIV status and make them feel stressed [32]. Scaling up the single tablet regimen and reducing dose frequency could thus help to improve mental health among PLWH on ART.

Relatively few participants reported an improvement in their sex-partner relationship, but for those who did, it was protective against depressive and anxiety symptoms. Health communication messages should therefore be disseminated to PLWH and their sexual partners emphasizing that viral suppression by ART greatly reduces the risk of transmitting HIV through sex [1, 2]. This may remove concerns about resuming sexual behaviour and improve intimacy between sexual partners. Most participants regained self-confidence during ART, and this was also protective against depressive symptoms. Studies have shown that ART makes people feel ready to return to normal life [31]. Our findings demonstrate the clinical benefits of the new “treat all” policy, as more than 80% of PLWH experienced improvements in physical health, which contributed to improved mental health, and nearly 90% experienced a rise in CD4 cell count.

However, our study has some limitations. First, as participants were recruited by non-probabilistic sampling, the findings may not be generalizable to all PLWH in China. Second, due to attrition bias, the prevalence of mental health problems at Month 12 may be underestimated, as those who did not complete both follow-up surveys might have worse psychosocial statuses. Selection bias may exist, as we could not obtain the characteristics of those who refused to join the study. Third, the positive and negative experiences related to ART were self-developed and had not been validated. Further studies are encouraged to focus on ART-related experience exhaustively. Fourth, it’s not clear whether the association between ART experiences and mood symptoms persists in a long term, as all the participants were new to ART in current study. Fifth, substance use may play a role in mood outcomes but it was not examined in the study. Moreover, the results were self-reported and thus subject to social desirability bias.

## Conclusions

In conclusion, our study verified the benefit of ART on mental health status of PLWH and highlighted the role of ART-related experiences in mental health status improvement. Tailored interventions targeting specific experiences such as to reduce side effects, inconvenience in daily life due to treatment, and to enhance partner relationship are warranted in future.

## Data Availability

The datasets used and/or analysed during the current study are available from the corresponding author on reasonable request.

## Abbreviations

ART: Antiretroviral therapy
PLWH: People living with HIV
UNAIDS: The Joint United Nations Programme on HIV/AIDS
PHQ-9: The Patient Health Questionnaire
GAD-7: The Generalized Anxiety Disorder Scale
MSPSS: The Multidimensional Scale of Perceived Social Support
CSSSs: The Chinese Courtesy Stigma Scales
